# Hysterectomy for the Management of Menstrual Hygiene in Women With Intellectual Disability. A Systematic Review Focusing on Standards and Ethical Considerations for Developing Countries

**DOI:** 10.3389/fpubh.2018.00338

**Published:** 2018-11-28

**Authors:** Horacio Márquez-González, Edith Valdez-Martinez, Miguel Bedolla

**Affiliations:** ^1^Hospital Infantil de México “Federico Gomez”, Mexico City, Mexico; ^2^Congenital Heart Disease Department, Cardiology Hospital, Centro Medico Nacional Siglo XXI, Mexican Institute of Social Security, Mexico City, Mexico; ^3^Health Research Council of the Mexican Institute of Social Security, Mexico City, Mexico; ^4^Policy Studies Center of the College of Public Policy, University of Texas at San Antonio, San Antonio, TX, United States

**Keywords:** intellectual disability, hysterectomy, menstruation, hygiene, developing countries

## Abstract

**Background:** Menstruation poses particular challenges for women with intellectual disability (ID). In low-and middle-income countries, where these women do not have access to facilities and resources for adequate menstrual care, hysterectomy could be considered as an ethically acceptable procedure. We conducted the first systematic review to identify what constitutes best practice for menstrual hygiene in women with ID and explored the perspectives of actors involved in the hysterectomy decision.

**Methods:** Theory-informed mixed-method thematic systematic review with theory development.

**Results:** Eleven ethical guidelines and 17 studies were included. Respect for autonomy and the patient's best interest were the criteria to determine what constitutes best practice. The actors' values and attitudes expressed some dimensions of existing inequities. In low-and middle-income countries, the main concern of parents was the difficulty to train their daughters about menstrual hygiene. Parents (mothers in particular) also expressed the feeling of being excessively burdened, and complained about the limitations of their support networks. Doctors perceived hysterectomy as a safe procedure and a solution for women with ID, whose menstrual hygiene is problematic. In general, the more severe or profound the level of ID, the more likely the interested parties advocated for a hysterectomy. The women with ID perceived their menstruation as a negative experience. Hence, the three parties supported hysterectomy for menstrual hygiene. Parents and doctors considered informed consent or assent (from the women with ID) as necessary and achievable.

**Conclusion:** The international ethical guidelines suggest that non-therapeutic hysterectomy in women with ID should not and ought not to be recommended as routine and appropriate method to cope with menstrual hygiene even if it is technically safe. Although hysterectomy to cope with menstrual hygiene is still a live issue in high-, middle-, and low-income countries, in high income countries it is performed with authorization from the Court; whilst in low-and middle-income countries there is not an active involvement of the State, or financial or training support for women with ID and their carers. Hence, in low-and middle-income countries there is an urgent need to develop and enact policies and statutes in this area of public health and clinical practice.

## Introduction

Intellectual disability (ID) has been defined by the World Health Organization as “a state of arrested or incomplete development of mind, which means that the person can have difficulties understanding, learning, and remembering new things, and in applying that learning to new situations” (1). ID is also known as learning disability, learning difficulty, intellectual impairment, developmental disability, and formerly as mental retardation or mental handicap or mental deficiency. It includes genetic disorders or biological abnormalities (Down syndrome, Fragile X syndrome, autism spectrum disorders, metabolic disorders, etc.) and non-genetic disorders (complications during pregnancy and delivery, encephalitis, meningitis, etc.).

Menstruation, like other natural human physiological processes, requires proper hygiene. Nazli and Chavan (2), Ranganath and Rajangam (3), Scola and Pueschel (4), among others, show that most women with ID have a normal menstrual pattern, similar to those of their peers without ID; i.e., regular menstrual cycles (length of cycles and flow), and no premenstrual or menstrual symptoms. Whereas, Joshi and Joshi (5), Burke et al. (6), Quint et al. (7), among others, show that women with ID have more likelihood to experience a variety of behavioural difficulties and physical symptoms before and during menstruation. In a review of primary studies about menstrual hygiene and menstrual health, Chandra-Mouli and Patel (8) highlights the vague measures used to describe the menstrual experiences of adolescents, the use of the terms premenstrual syndrome and dysmenorrhoea loosely, small sample sizes, and self-report predominance.

Menstruation entails concerns about menstrual hygiene (e.g., dealing appropriately with menstrual flow and pain, and the associated behavioural difficulties) and reproductive issues for and amongst the women with ID, their carers, and the health system, particularly in low-and middle-income countries (9). With advances in hysterectomy techniques, definitive amenorrhea may be seen as the ideal way for dealing with the problems of menstrual hygiene, not only medical, but social conditions (e.g., poverty, absence of social support networks). Definitive amenorrhea would eliminate carer burden, parental distress, unwanted pregnancies, potential medical problems such as infections, etc. Yet, the appeal to non-therapeutic hysterectomy to achieve menstrual hygiene, and in turn contraception, raises a host of ethical/bioethical, legal, and medical concerns. It is nevertheless true that in low-and middle-income countries it often continues to be practiced. Within this context, there exists an intense debate around the indication of non-therapeutic hysterectomy for menstrual hygiene, in women with ID. At least four sources of the controversy are evident.

The first lies in facts and probabilities; for example, there are those who say that menstruation in women with ID affects the welfare of the women themselves and has very significant financial, relational, and emotional consequences for the rest of the family (10, 11). Others say that educational and behavioural modification programs to address the problems associated with the menstrual-care needs of this group of women are not available in low-and middle-income countries, like Mexico (12, 13). Opponents cite the Nuremberg Laws (14) and present the slippery-slope argument: if rules permitting non-therapeutic hysterectomy in women with ID become public policy to avoid social and family burdens, the same logic can be extended to other populations of feeble or seriously ill patients who also are financial and emotional burdens on family and society; for example: girls and young women without ID but with significant physical long-term difficulties.

The second source of the controversy has to do with the moral status of the women with ID. Some claim that a woman with ID has significant limitations in both intellectual functioning and in adaptive behaviour to manage her estate, to raise children, and like; therefore this woman, and women like her, has a “lower” moral status, and less rights, that allows for a non-therapeutic hysterectomy (11, 15). Others disagree and claim that women with ID have the same moral status and rights as women without ID (16, 17). In fact, this controversy has to do with the model with which the problem is approached. For the medical model a disability always lowers the moral status of the person, whereas, when the disability is approached from a social model, it is neither good nor bad and there is room to see if the disabled person is still able to exercise her right to self-determination (18).

The third source of controversy arises when decision makers (parents, physicians, society at large) place value on different interests. For instance, some argue that parents' rights over their daughter with ID extend to the request of a hysterectomy for menstrual hygiene (19). Others disagree and highlight the fact that the rights of the women with ID cannot be superseded by the interests of their parents/carers (20). Hence they limit the extent of parental decision-making (21).

The fourth source of controversy arises from differences in cultural attitudes and beliefs, and social norms; for example: in Taiwan (22) menstruation is perceived by mothers as a natural event and surgical procedures are not approved for menstrual suppression. In Australia and United Kingdom (21), even if the woman's parents and doctors support hysterectomy, it cannot be performed without the sanction of the Court. In South-Africa and India (21, 23), for example, hysterectomy to address the concerns of menstrual hygiene and unwanted pregnancy is accepted. In México, non-therapeutic hysterectomy is un-lawful; nonetheless, it has been offered over many years as the preferred surgical procedure for menstrual hygiene and sterilization in adolescents with ID (24), whose parents or guardians have sought this service and when psychiatric and/or neurological and /or paediatric consultation concurred with the parents. Differences in cultural attitudes and beliefs are reminiscent of the religious practices and beliefs related to menstruation (25); for instance: in Judaism and Hinduism menstruating women are considered “ritually unclean”; in Islam all menstruating women are considered “ritually impure”; whereas in Buddhism and Sikhism menstruation is seen as a “natural physical excretion,” even Sikhism condemned the practice of treating women as impure while menstruating. All of these groups have the evidence necessary to present good arguments in support of their positions.

The purpose of this paper was to review the literature and to explore these issues in great depth. The review is important because scoping the literature found no relevant systematic review of the topic; hence, a high quality theory-informed synthesis of evidence is indispensable. The review is both timely and important, and the evidence is needed to better understand the support that women with ID, carers and practitioners need to address the problem associated with menstrual-care needs. Furthermore, it is hoped that elucidation of these issues may start a process of discussion by which they can be resolved by policy makers in low-and middle-income countries, to the ultimate benefit of those affected.

## Methods

The following objectives were defined to help explore the search and synthesis of evidence:

- To explore the evidence about the experiences and attitudes of women with ID, parents/carers, and healthcare workers toward non-therapeutic hysterectomy (for menstrual hygiene).
- To identify the medical benefits and harms of non-therapeutic hysterectomy in women with ID.
- To determine what constitutes best practice within the context of selected ethical guidelines concerning non-therapeutic hysterectomy in women with ID.

### Review design

**Figure 1:**
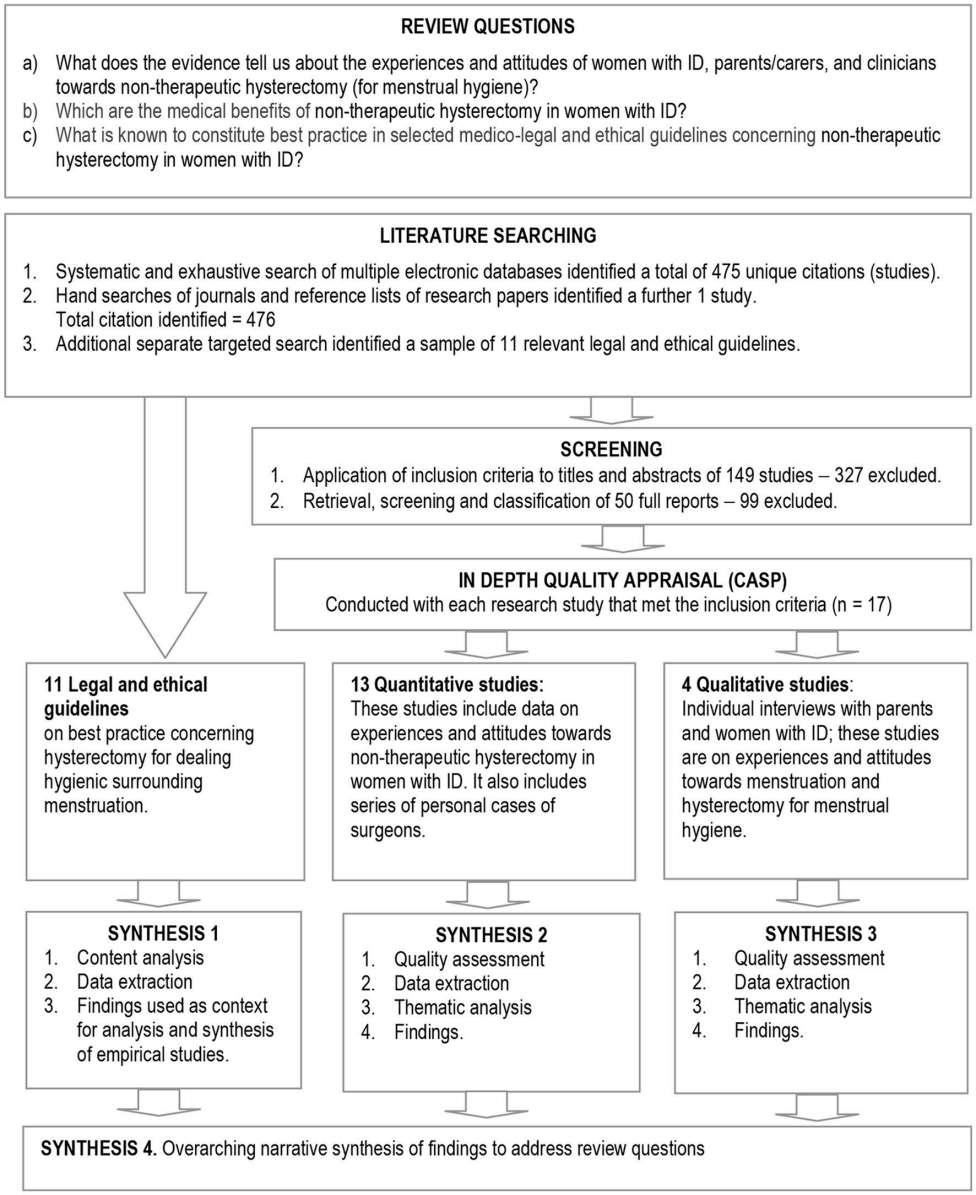
Flow diagram of the review design and process.

Initial scoping of the literature showed that a mixed-method design was most appropriate to address the objectives. The Evidence for Policy and Practice Information (EPPI) and the EPPI Centre Guidance on synthesis of mixed-method evidence (26) was selected. The EPPI approach was adapted to enable quality screening, and analysis and synthesis of evidence within three separate synthesis streams: ethical guidelines, quantitative and qualitative studies of any type (Figure 1). Mixed-method studies were not found. The analysis of ethical guidelines was designed to address objective “c”; the synthesis of other quantitative and qualitative studies (streams 2 and 3) was designed to explore objectives ”a” and “b.” Findings from streams 1 to 3 were then brought together in an overarching synthesis to address the review objectives. Further description of the synthesis processes can be found in the section on data extraction and synthesis.

### Search strategy

A simple search strategy as advocated by Flemming and Brings (27) was used to locate studies and is summarized in the SPICE table (Table 1) (28) defining the setting, perspective, phenomenon of interest, comparisons, evaluations and methodological approaches. The search strategy was based on key concepts of interest from the objectives. The search terms used included the recognised Medical Subject Heading (MESH). The search terms used included: wom^*^ OR adolescen^*^ OR youth^*^ OR teen^*^; doctor OR clinician; parent^*^ OR family. These keywords were combined with: mental^*^ retarda^*^ OR mental^*^ disab^*^ OR intellectual disab^*^ OR development^*^ disab^*^ OR learn^*^ disab^*^; hysterectomy AND mens^*^ OR menstruation OR menstrual hygiene; ethic^*^ OR perception^*^ OR approach^*^ OR experience^*^ OR coping.

**Table 1:** SPICE search strategy.

**Setting**	**Perspective**	**Phenomenon of interest**	**Comparison**	**Evaluation**	**Methodological approach**
Clinical practice and ethical setting	Women with ID	Approaches and experiences toward menstruation and hysterectomy for menstrual hygiene.	Key stakeholders perspectives (women with ID and their parents/carers, and healthcare professionals). Ethical guidelines.	Content analysis of guidelines.Comparative and thematic analysis and narrative synthesis of quantitative and qualitative studies.	Quantitative Qualitative Guidelines

PubMed, Scopus, DOAJ, Web of Science, EMBASE, and Health Virtual Library (LILACS) were searched electronically. In addition, the Web sites of United Nations, UNESCO, World Health Organization, Word Medical Association, International Federation of Gynaecology and Obstetrics, Council of Europe, American College of Obstetricians, and Gynaecologists, and Mexican law–as an instance of low-and middle-income countries, were also searched. No publication date restriction was imposed on searches as this is an original review. The electronic searches were supplemented with hand searching of key journals, such as: Journal of Intellectual Disability Research and Salud Publica Mexico.

Inclusion and exclusion criteria: Studies eligible for inclusion were those quantitative or qualitative research studies of any type that reported experiences, attitudes, and understandings of women with ID, their parents/carers and healthcare providers, regarding hysterectomy for menstrual hygiene. As well as ethical guidelines, the search for key selected ethical guidelines was purposively limited in order to extract key tenets of international guidelines to serve as context for interpreting evidence from published studies. Published and unpublished studies reported in English and Spanish language. It excluded studies if they used a sample of mixed respondents (e.g., women with different degree of ID and women with ID and their carers) where results of women with ID cannot be clearly separated. Conference abstracts, editorials and commentaries were not included.

### Search outcome

The initial electronic searches identified 475 citations (Figure 1). From these citations, the titles and abstracts were reviewed by FR and checked by EV, of which 49 citations required a full document screen to determine if they met the inclusion criteria. It was unclear whether these studies targeted hysterectomy for handling hygienic surrounding menstruation in women with ID. Hand searching identified 1 further research studies that required a full document screen. Of 50 full document screened, 17 studies met the inclusion criteria and were included in this review. Eleven guidelines established criteria to determine which norms should govern “best practice” regarding hysterectomy for menstrual hygiene.

### Quality assessment

Studies were appraised within each stream separately using the relevant versions of the Critical Appraisal Skills Programme tool (CASP) (29). Any disagreements were resolved through discussion between reviewers. No studies were excluded on the basis of quality. No study had a fatal flaw (the threshold for exclusion). Guidelines were not appraised critically.

### Data extraction and synthesis

EV and HM independently extracted and summarized evidence by stream in tables and templates adapted from National Institute for Health and Clinical Excellence (NICE) guidance (30). Guidelines were subject to content analysis (31) and key guiding principles underpinning ethical non-therapeutic hysterectomy were extracted and summarized in Table 2. Key ethical principles were then used as context to define best practice when interpreting evidence. Quantitative and qualitative (no mix-method research studies were found) streamed and extracted data were summarized in Table 3. Data extraction and any queries were resolved by consensus between EV and HM.

**Table 2:** Summary table of international laws, guidelines, and regulations upon hysterectomy and menstrual hygiene in women with intellectual disability.

**Organization or country**	**Law [L] Guideline [G] Regulation [R]**	**Specific provision**
UNITED NATIONS	[G] The Universal Declaration of Human Rights (1948) (32)	Article 2 “Everyone is entitled to all the rights and freedoms set forth in this Declaration, without distinction of any kind…”
	[G] The United Nations Convention on the Rights of the Child (1990) (33)	Article 2 (1) “…ensure the rights set forth in the present Convention to each child… irrespective of… disability or other status.” Article 2 (2) “…appropriate measures to ensure that the child is protected against all forms of discrimination on the basis of… beliefs of the child's parents, legal guardians, or family members.” Article 24 (3) “…appropriate measures with a view to abolishing traditional practices prejudicial to the health of children.”
	[G] Convention on the elimination of all forms of discrimination against women (CEDAW) (1979) (34)	Article 3 “…appropriate measures, including legislation, to ensure the full development and advancement of women, for the purpose of guaranteeing them the exercise and enjoyment of human rights…”
	[G] Convention on the rights of persons with disabilities (2006) (35)	Article 2 “Discrimination on the basis of disability means any distinction, exclusion or restriction on the basis of disability which has the purpose or effect of impairing or nullifying the recognition, enjoyment or exercise, on an equal basis with others…” Article 7 (2) “In all actions concerning children with disabilities, the best interests of the child shall be a primary consideration.” Article 12 (4) “…safeguards shall ensure that measures relating to the exercise of legal capacity respect the rights, will and preferences of the person, are free of conflict of interest and undue influence…” Article 16 (2) “…appropriate measures to support for persons with disabilities and their families and caregivers.” Article 23 (c) “Persons with disabilities, including children, retain their fertility on an equal basis with others.”
UNESCO	[G] Puberty, education, and menstrual hygiene management (Good policy and practice in health education) (2014) (36)	Page 15 **“**…Educators must ensure the content and methodology are developmentally appropriate for learners with learning disabilities….”
The International Federation of Gynaecology and Obstetrics (FIGO)	[G] Ethical issues in obstetrics and gynaecology by the FIGO committee for the study of ethical aspects of human reproduction and women's health (2012) (37)	Paragraph 2. “Women with severe intellectual disability…may require a surrogate in order to make best interests decisions about their hygiene and health.” Recommendation 5 **“**If a woman has a disability so severe as to understand her menstruation…or if she does not have the capacity to maintain adequate personal hygiene during menstruation, it is ethically as well as medically and prudently indicated to seek a medical or surgical option and the least invasive possible.”
United States of America	[G] The American College of Obstetricians and Gynaecologists (2016) (38)	“When the obstetrician-gynaecologist receives a request for menstrual suppression, it is important to assess the reasons for the request… to determine if menses… is a matter of preference of the patient or convenience of the caregiver.” “Hysterectomy for the purpose of cessation of normal menses may be considered only after other reasonable alternatives have been attempted.”
Council of Europe	[G] Safeguarding adults and children with disabilities against abuse (2003) (39)	“Major and irreversible interventions such as sterilization of a mentally incapacitated child or adult need to be located in the courts to safeguard against excessive family control, especially around controversial issues such as independent living, sexuality, childbearing and risk-taking.”
Mexico	[L] General Law for the Inclusion of Disabled Persons (2015) (40)	Article 7 (X) **“**Bring about programs for the orientation, education and sexual and reproductive rehabilitation of disabled persons and their families.” Article 21 (II) “Establish programs for the delivery of social assistance for disabled, low income persons…”
	[L] Federal Law to prevent and eliminate discrimination (2016) (41)	Article 5. “…It will not be judged as discrimination that distinction which is based on reasonable, proportional, and objective criteria whose intention is not to deny any rights.”
	[L] General Law about the rights of female and male children and adolescents (2014) (42)	Article 50 (V) **“**To develop preventive health care and the orientation of those who are custodians…in sexual and reproductive matters.” Article 50 (XIII) “To prohibit, to punish and to eradicate forced sterilization of male and female children and adolescents and any form of obstetric violence.”

**Table 3:** Summary table of included studies.

**References**	**Objective**	**Study design**	**Participants**	**Setting**	**Results**	**Methods/Quality**
Wheeless (43)	To explore current legal guardians' opinions toward hysterectomy, after the time of the operation.	Cross-sectional, questionnaire-based study. Structured questionnaire. Face-to-face interviews.	Legal guardians of 92 women with ID. Level of ID is not reported. Women mean age 23 years (range: 10-48 years) at time of operation. 82.6% nulliparous. Abdominal hysterectomy was offered and performed between the years 1950 and 1973.	The Johns Hopkins University, School of Medicine. USA	95% requested hysterectomy. 86% felt relived of the care of the menstrual period. 93% felt that the operation had no abnormal effect on the patient. 96% would make the same decision (about hysterectomy).	Questionnaire validation is not reported. Trained interviewers are not reported. No sample size estimation No probabilistic sampling Missing data given the retrospective nature of the study. Different guardians over the period of study.
Chamberlain et al.(44)	To review the experiences and attitudes of primary carers relative to menstruation and contraception.	Cross-sectional, questionnaire-based study. Structured questionnaire. Face-to-face interviews.	69 parents (58 were mothers) of 87 women with ID (41 mildly; 23 moderately, 23 severely). Women mean age 16.7 ± 2.7 (SD) years. No parity declared. Parents of mildly ID sought contraception first. Parents of severely ID sought menstrual suppression first.	The Cincinnati Adolescents Clinic. USA	Among the women with severe ID, who were menstruating, 15 of the 17 mothers complained of difficulty in training their daughters to handle their menses. 41% and 27% of parents of moderately and mildly ID patients had difficulty teaching their daughters menstrual hygiene.	Questionnaire validation is not reported. Trained interviewers are not reported. No sample size estimation No probabilistic sampling Missing data given the retrospective nature of the study.
Passer et al.(45)	To explore parental attitudes toward sterilization.	Cross-sectional, questionnaire-based study. Structured questionnaire. Face-to-face interviews.	69 parents (58 were mothers) of women with ID (33 mildly; 16 moderately, 20 severely). Women mean age 16.7 ± 2.7 (SD) years. No parity declared. Parents of women with severe ID requested for hysterectomy first.	The Cincinnati Adolescents Clinic. USA	Interest in sterilization especially correlated with increased severity of ID (*p* < 0.05) and with difficulty teaching menstrual hygiene (*p* < 0.05). Parents seek sterilization, not for fertility control, but rather for elimination of menses.	Questionnaire validation is not reported. Trained interviewers are not reported. No sample size estimation No probabilistic sampling.
Carlson and Wilson (46)	To detail information about decision making on menstrual management on behalf of women with learning disabilities and high support needs.	Cross-sectional, questionnaire-based study. Semi-structured face to face interviews.	30 mothers of 30 women with an ID and high support needs. Level of ID is not reported. Women's ages ranged from 13 to 28 years. Parity not declared. 14 had hysterectomy at an average age of 14 years. 3 of them had premenarcheal hysterectomy. Mothers of 8 daughters (5 premenarcheal, 1 menstruation suppressed through pill and 2 menstruating) were considered hysterectomy. Mothers of 6 menstruating daughters not considering menstrual elimination.	Intellectual Disability Services, QDFSAIA, Australia	Menstrual elimination was preferred. The most frequently reasons given: Menstrual hygiene, Concerns about pregnancy, Management of physical discomfort or emotional changes. ‘Heavy' menstrual flow. Influences in the decision: Mother's experiences and her attitudes toward menstruation. Perceiving daughter as a perpetual child. Attending Doctors and Family.	Questionnaire validation is not reported. Used of trained interviewers are not reported. No sample size estimation. No probabilistic sampling. Potential recall bias. No specification of ID levels.
Rodgers (47)	To describe the experiences of menstruation of women with ID.	In-depth, face-to-face interviews. (qualitative study)	21 women with ID (“it might be assumed to have mild or moderate”). No women's age reported. Parity not declared 2 reported hysterectomies: one for menstrual pain and one for heavy periods.	South West of England UK	Many wished they did not have periods. Their main concerns were: pain, dealing with menstrual flow and embarrassment. Women with hysterectomy supported the procedure and advocated it for other women. They did not understand the relationship between menstruation and fertility.	No data saturation reported. Inductive approach of data collection. Grounded theory methodology. No stratification by ID.
Dizon et al.(48)	To review the primary carers' concerns and preferences with regards to menstruation and contraception.	Cross-sectional, retrospective chart review of hospital records from 1998 to 2003. .	The charts of 72 women with cognitive disability (no degree of ID declared). Mean age 12.3 years (range: 8-17 years) at the first consultation. 31 of the 72 were premenarcheal. Parity not declared Menstrual hygiene problem was the primary concern in 65 (90%) families. Menstrual hygiene and contraception issues in 4 families. Contraception in 3 families.	The hospital for Sick Children. Toronto, Canada	67% requested for menstrual suppression. 11% requested for permanent sterilization. 42% accepted Depo-Provera as method of menstrual suppression. 1 underwent hysterectomy.	Questionnaire validation is not reported. No sample size estimation No probabilistic sampling Missing data given the retrospective nature of the study. No stratification by ID level.
Rodgers (49)	To know what help is given to the women in relation to menstruation.	Cross-sectional, questionnaire-based study. Structured questionnaire. Postal survey.	Carers of 452 women with ID (66 mild; 156 moderate; 217 severe o profound). Mean age 36 (range: 14-55). Parity not declared 11 had had hysterectomy.	ID registers in 2 areas of England. UK	A range of people contribute to teach how manage menstrual care. The most common groups involved were mothers and residential staff. Carers were given considerable amounts of assistance with menstrual care. The level of independent menstrual care was not statistically associated with the level of ID nor with age.	Questionnaire piloted No sample size estimation No probabilistic sampling Missing data given the retrospective nature of the study.
Stansfield et al. (50)	To describe a consecutive series of referrals to the Official Solicitor.	Cross-sectional, retrospective case note study from 1988 to 1999.Information obtained from clinical and legal notes.	In 72 women with ID, sterilization was requested (11 mild; 27 moderate; 28 profound; 6 unclassifiable). Mean age 21.8 years (range: 12-35 years) at initial contact. 2 premenarcheal 10 with previous pregnancy.	Official Solicitor's Office. England and Wales. UK	39 cases went to the court. 10 requested for surgery because of menstruation difficulties (as career). 7 were non-therapeutic hysterectomies, for menstrual difficulties, approved by the court.	Pilot test of data collection, No sample size estimation. No probabilistic sampling. Missing data given the retrospective nature of the study No stratification by ID level
Zacharin et al. (51)	To assess the impact of menstruation on the lives of adolescents and their families.	Cross-sectional, questionnaire-based study Structured questionnaire. Telephone interview or Postal survey.	Parents or carers (96 were mothers) of 103 women with cerebral palsy (42 mild, 12 moderate, 48 severe). Women mean age 15 years (range: 12-18 years). 24 were premenarcheal Parity is not declared None of them with hysterectomy.	The Royal Children's Hospital. Melbourne, Australia	An inverse relationship between the carer being happy with the current management of the menses and the severity of the menses (*p* = 0.034). No significant correlation between the happiness of parents with the current management and their child's level of disability (*p* = 0.069).	Questionnaire validated Trained interviewers are not reported. No sample size estimation. No probabilistic sampling
					The severity of the child's disability was significantly correlated (*p* = 0.001) with the likelihood that the families had sought medical advice.	
Chou and Lu (52)	To explore decision-making regarding hysterectomy	A semi-structured face-to face interviews (qualitative study)	Mother of a woman of 20 years old with severe ID. Nulliparous Never married With hysterectomy	Hsinchu City,Taiwan	The mother saw the hysterectomy as the only way to solve the menstrual care problem (as carer). The mother's decision was initiated and suggested by health professionals. The woman with ID was not involved in the decision or informed.	Volunteer mother No data saturation reported. Inductive approach to data collection. Constant comparative method for analysis. Thematic analysis.
Chou and Zxy-Yann (53)	To explore mothers' experiences of managing their daughters' menstruation.	A semi-structured face-to face interviews (qualitative study)	12 mothers of 13 women with ID (1 marginal, 5 moderate, 7 profound) Women age ranged from 18-43 years old. No parity declared None of them with hysterectomy	Hsinchu City, Taiwan	Hysterectomy or use of medications to cease menstrual bleeding was never considered by the mothers. Support networks were limited and mothers developed their own strategies for managing their daughters' menstruation. The reasons the mothers did not approve surgical procedures were related to cultural perceptions of the body and how it functions.	No pilot of interview schedule. The study results were limited to mothers recruited voluntarily whose daughters were menstruating. No data saturation reported. Thematic analysis
Thapa and Sivakami (13)	To explore experiences of adolescents and their carers surrounding menstrual hygiene management.	In-depth, face-to-face interviews (qualitative study)	23 mothers of 23 adolescents with ID (20 moderate and 3 severe). Adolescents' ages ranged from 11 to 19 years. All had menarche. Parity not declared. 3 adolescents (2 moderate and 1 severe ID) had hysterectomy for menstrual hygiene. All belonged to a high income bracket. The 3 mothers expressed high level of satisfaction with their decision. No complications 3-4 years after surgery.	Educational institutions for children with ID in Delhi, India	Mothers were often the only external source of information, guidance & training. All mothers (without skills to train & guide) felt solely responsible for their daughters' menses. All adolescents were without information or training before menarche. The adolescents' refusal to accept use of pads interpreted as inability to accept or understand. Some mothers did not attempt to train their daughters because they felt it would not help.	No data saturation reported. Used of trained interviewers are not reported. Grounded theory methodology. No specification of type of data analysis.
Perrin et al. (54)	To describe 10 cases of hysterectomy	A longitudinal, retrospective chart review of hospital records from1970 to 1973.	The charts of 10 women with ID (with different, unspecified, levels of ID). Age ranged from 14-19.5 years old. Parity was not reported. 9 unable to cope with menstrual hygiene independently. 1 had severe menorrhagia. 10 abdominal hysterectomies.	Children's hospital of Michigan, Detroit, USA	No intra-operative or postoperative complications were reported. No histopathologic post-operative reports. Parental satisfaction was high. Mothers were greatly relieved by the elimination of menstruation responsibility.	Questionnaire validation is not reported. No sample size estimation. Missing data given the retrolective nature of the study. Long-term follow-up was not conducted Methodology to assess satisfaction is not reported. No specification of ID levels.
Kaunitz et al. (55)	To describe five cases of hysterectomy.	A longitudinal, retrospective chart review of hospital records over 1985.	The charts of 5 women with profound ID. Age ranged from 13-27 years old. All nulliparous 2 no menarche Inadequate menstrual hygiene 4 vaginal and 1 abdominal hysterectomies.	Jacksonville University Hospital. Florida's indigent community. USA	No intra-operative or postoperative complications were reported. Histopathologic reports showed uteri healthy. All mothers expressed satisfaction with the surgical results.	Questionnaire validation is not reported. No sample size estimation. Missing data given the retrolective nature of the study. Medium or long-term follow-up was not conducted. Methodology to assess satisfaction is not reported.
Sheth and Malpani (56)	To describe 60 cases of vaginal hysterectomy.	A longitudinal, retrospective chart review of hospital records from 1968 to 1987.	The charts of 60 women with severe ID. Age ranged from 13 to 31 years old. All had an intact hymen. All nulliparous Poor menstrual hygiene or irregular menstruation. All vaginal hysterectomies.	King Edward Memorial Hospital. And private practice. Bombay, India.	No intra-operative complications. No significant post-operative complications. No histopathologic post-operative reports. All parents expressed satisfaction with the surgical results.	Questionnaire validation is not reported. No sample size estimation. Missing data given the retrolective nature of the study. Medium or long-term follow-up was not conducted.
Piña-Perales et al. (24)	To describe six cases of hysterectomy.	A longitudinal, retrospective chart review of hospital records from 2004 to 2011.	The charts of 6 of 11 women with ID (2 moderately, 4 severely). Age ranged from 12-17 years old. Parity was not reported. Poor menstrual hygiene (*n* = 4) or irregular menstruation (*n* = 2). 5 vaginal and 1 abdominal hysterectomies.	Paediatric Hospital of the National Medical Centre, S. XXI. Mexico.	1 lesion of bladder. No postoperative complications 3 months after surgery. No histopathologic post-operative reports. Patients' families expressed quality-of-life improvement both for patient and for their carers.	Questionnaire validation is not reported. No sample size estimation. Missing data given the retrolective nature of the study. Long-term follow-up was not conducted. Methodology to assess quality-of- life is not reported.
Chalermchockcharoenkit et al. (57)	To evaluate both the safety of laparoscopic hysterectomy and satisfaction of the carers.	A longitudinal, retrospective chart review of hospital records from 2004 to 2010. Structured telephone interviews with parents or carers.	The charts of 74 women with ID (13 mild, 38 severe, 23 unspecified). Women mean age 14.9 ± 4.2 (SD) years. Parity was not reported. Poor menstrual hygiene was the main issue for 97% women, and Inappropriate behaviour for 3% women. 74 laparoscopic hysterectomies.	Siriraj Hospital, Bangkok, Thailand	No major intra-operative or post-operative complications were reported. Only 2 uteri had endometrial polyps. The remainder 72 (97%) histopathologic reports showed uteri healthy. All carers expressed high level of satisfaction with the surgical results, 3 months after surgery.	Questionnaires validation is not reported. Trained interviewers are not reported. Sample size was estimated. Missing data given the retrolective nature of the study. Long-term follow-up was not conducted.

*ID, Intellectual disability; QDFSAIA, Queensland department of Family Services and Aboriginal and Islander Affairs*.

It was impossible to perform a meta-analysis as we did not find any clinical trials and observational studies were heterogeneous and did not permit statistical pooling. Therefore, a thematic synthesis approach (58) was used to synthesise evidence from streams 2 and 3. We undertook a thematic synthesis with 17 studies (by stream: 13 quantitative studies, 4 qualitative studies, and 0 mixed method studies). It is important to note that the process of coding primary studies was undertaken in an inductive way. We identified decision-makers and stakeholders involved in the problem, unpicked their values, their beliefs and their explanations and justifications to be used for choosing non-therapeutic hysterectomy, and identified key alternatives and objectives and information available.

The analysis process required each study to be read repeatedly to ensure that all concepts were integrated and the relationships between the concepts of each study were explored. We used the notion of first order, second order and third order constructs to analyze and reinterpret the studies (58). First order constructs are insights offered by participants in the original study. All participant quotations that were paraphrased by the original researchers were extracted as first order constructs. Second-order constructs are the interpretative themes that were developed by the original researchers from the first order constructs. We described and listed the themes reported by authors of each original study and made a note of the number of studies that contributed to each theme. Third order constructs were derived from the synthesis of evidence across multiple studies. We developed third-order constructs by analyzing the second-order constructs to identify new, common themes that emerged from our inductive analysis to address the review objectives. Data entry and analysis were performed using Atlas/ti version 7 computer software.

### Interpreting the entire dataset and developing a new theoretical framework

**Figure 2:**
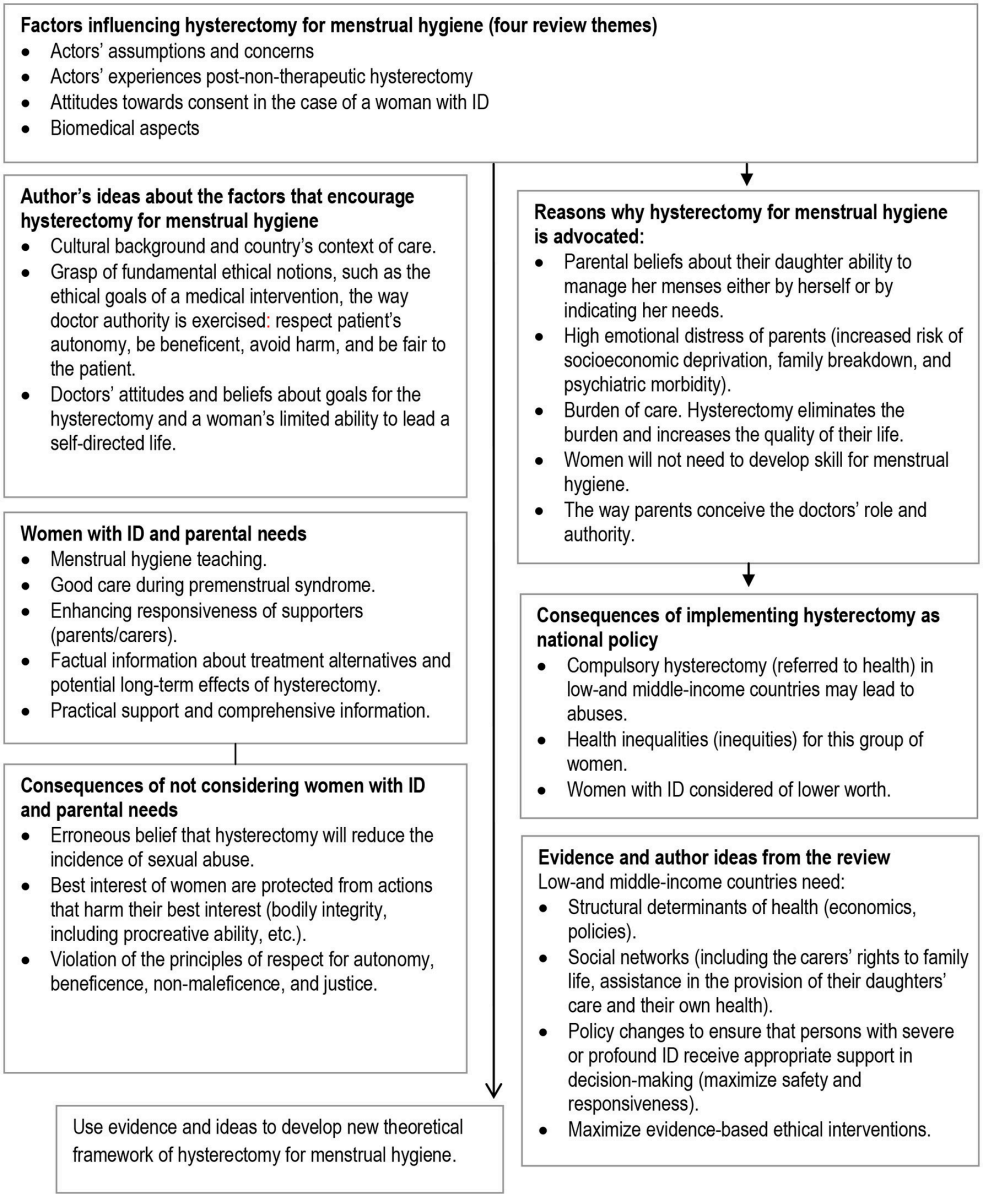
Factors, ideas and hypothesis generation on performing hysterectomies for menstrual hygiene in women with ID–from relevant actors and author perspective.

**Figure 3:**
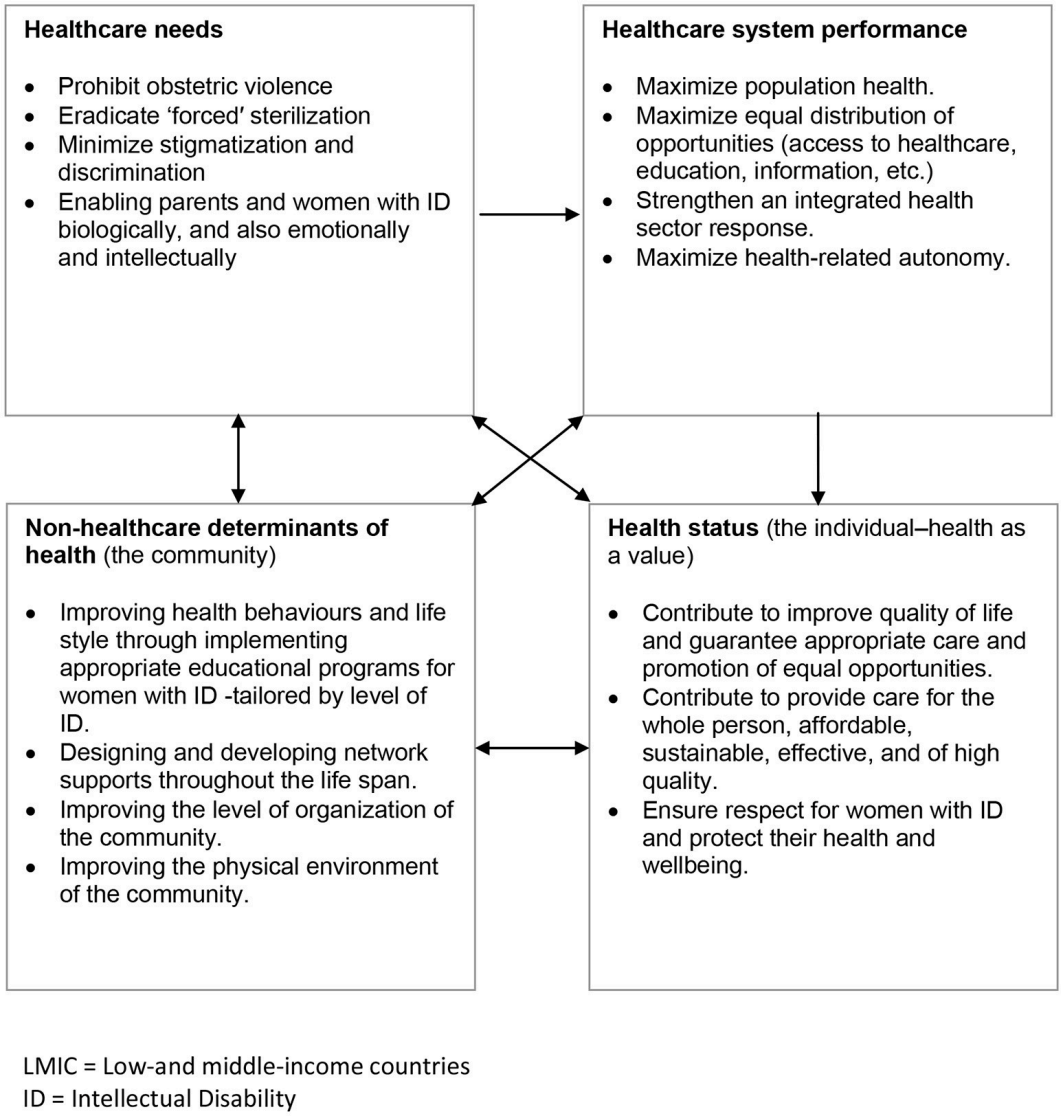
Theoretical framework of representative influences and their relationships to the interest not to perform hysterectomy for menstrual hygiene as a public heath polciy in LMIC (an arrow means “influences”). Based on Edwards W, Miles RF, Winterfeldt DV. Advances in decision analysis. From foundations to applications. New York: Cambridge University Press; 1993.

Finally, we synthesised evidence from the three streams in an overarching fourth narrative synthesis (Figure 1). We juxtaposed key tenets of best ethical practice in key selected guidelines (such as “best interests,” respect for autonomy, etc., described in Table 2) against the themes from 17 studies using a constant comparison technique (31) in order to see the extent to which these tenets are socially enforced. First level constructs (participant quotes) and second level constructs (primary research author interpretations) were translated into the 4 themes (third order constructs) to which we then added our own ideas and interpretations through ongoing engagement with the evidence and using our expert knowledge of the field to form hypotheses concerning what the evidence said about hysterectomy for handling hygienic surrounding menstruation in women with ID from relevant actors' perspectives (Figure 2). Several subsequent meetings helped the authors understand how women with ID, their parents/carers and their doctors shaped the goals, objectives and preferences in hysterectomy for menstrual hygiene and over time developed a new theoretical framework to help relevant actors and stakeholders the changes required of them and for guiding future research (Figure 3).

## Results

### Key tenets from ethical guidelines

The study of the most comprehensive publications on the substantive rights and obligations that protect the vulnerability of women with ID allowed the authors to identify 11 ethical guidelines (32–42) for hysterectomy and menstrual hygiene, in women with ID. The guidelines were purposively sampled, reviewed and then summarized to determine what constitutes best practice in accordance with commonly accepted medico-legal and ethical guidelines (Table 2).

#### What constitutes best practice in accordance with legal and ethical guidelines?

Best practice is that which is believed to be congruent with the best interest of women with ID. Best interest is the primary consideration among ethical guidelines. Best practice is also non-discriminatory and it promotes equity within the framework of health policies that prohibit obstetric violence; eradicate “forced” sterilization; and create appropriate educational programs for persons with learning disabilities and their families. The International Federation of Gynaecology and Obstetrics (FIGO) (37), in particular, acknowledges as best practice “the surgical option” for menstrual hygiene in women with profound or severe intellectual disability. Pertinent Mexican laws do not include specific measures for women with ID, although they are aligned with the international ethical guidelines' recommendations for the care of people with disabilities.

### Actors' assumptions and concerns

#### Parents/carers

In the studies included, the request for menstrual suppression or elimination because of hygiene concerns was the most common cited reason by the parents/carers, mainly with the women with severe ID (13, 44–46, 48, 50–52). For example, one survey revealed that 15 of the 17 mothers of the adolescents with severe ID complained of difficulty in training their daughters to handle their periods. All 17 mothers requested menstrual suppression first (44). Another survey, with 68 parents, disclosed that interest in menstrual suppression was especially correlated with increased severity of ID and with difficulty teaching menstrual hygiene (45). A postal survey, with 103 parents, showed an inverse relationship between the happiness of the carer with the current management of the menses and the severity of the menses. In addition, both the severity of the woman's disability and the level of impact of menstruation were significantly correlated with the likelihood that the families had sought medical advice (51). On that subject, a qualitative study that included the Taiwanese mother of a woman with severe ID, who underwent hysterectomy, saw hysterectomy as the only way to solve the menstrual-care problem. She felt that if she passed away earlier than her daughter, her husband and the siblings of the women in question should not be burdened with caring for a menstruating woman. She also described how her decision about hysterectomy was initiated and suggested by healthcare professionals (52). Likewise, three Indian mothers reported to have opted for a hysterectomy because they felt excessively burdened and did not want to pass on the “burden” to other family members (13). In line with these statements, the most frequent reason given by 30 Australian mothers for menstrual elimination was “care” (their daughter inability to change pads herself, and others not to accept this task); also, difficulties with their own menstruation, or a negative perception of menstruation, influenced their decision (46). In contrast, a qualitative study carried out with 12 Taiwanese mothers revealed that they never considered menstrual suppression medication or surgical elimination. They perceived menstruation as natural and good for the physical health of their daughters. The mothers in this study did not report management difficulties related to menstruation, even though their daughters had significant support needs and the support networks were limited (53). Similar statements appear in another qualitative study, where the 23 Indian mothers interviewed said that they handled their daughters' menstruation in their own way without much support from other sources, and without receiving any kind of specialized training. Some adolescents (from this group of mothers) lacked of training initiatives because their mothers believed that menses management training would not be of much; whereas the few adolescents who were self-reliant were repeatedly trained by their mothers, sometimes with the help of an older sister (13).

#### Women with ID

A single qualitative study, carried out in England, included interviews with women with mild or moderate ID (47), for all of them menstruation was a negative experience; their main concerns were menstrual pain, dealing with menstrual flow and embarrassment. These concerns were, in turn, closely associated with having to ask for painkillers (especially if it meant asking male carers); with the unpleasant messy bleeding; with the correct disposal of their sanitary towels/pads; and with the amount of money necessary to buy adequate pads. All of the concerns expressed by women were related to the idea of “embarrassment” that was, in turn, related to prevailing menstrual etiquette.

### Actors' experiences after non-therapeutic hysterectomy

#### Parents/carers

Only one quantitative study (43) explored the experiences with hysterectomy of the parents of 92 women with ID. It disclosed that 88 of 92 parents were completely satisfied with the procedure. Of these 92 parents, 86 responded that they felt that the operation had no abnormal effect on their daughter, and that it relieved the need for caring for the menstrual period. The remainder six parents stated that their daughter continued to have monthly cramps, headaches, and complained of abdominal pain; moreover, they (parents) felt that the lack of menstruation either affected adversely the female hormone system or did not relieve the system of “poison blood” each month.

#### Women with ID

Rodgers (47) on interviewing women with ID found that the two women who talked of having hysterectomy supported the procedure and said they would advocate it for other women. However, they also said that their doctors did not ask them about any plans to have children at first, and they did not know about the consequences of the medical intervention.

### Actors' attitudes toward informed consent from woman with ID

#### Parents/carers

A survey that included 69 interviews with parents shows that some of them (40%) felt only parents should give consent; while some others (40%) felt doctors should be involved in obtaining consent. These last group of parents stressed that “*doctors should have the ID person's best interest at heart*,” and “*be familiar with the case and family”* (45). In a qualitative study exploring decision-making, the mother of a woman with severe ID who underwent hysterectomy, stated: “*I did not tell her, I have no idea whether she can understand or not”* (52). Another study on decision-making, 26 of the 30 mothers interviewed said that only parents should make final menstrual management decisions. Menstrual elimination was perceived by most of them as a medical issue (46). A qualitative study with 23 mothers revealed that they think their daughters are “too naïve to understand” (13).

#### Doctors

All doctors (24, 54–57) reported that all hysterectomies in women with ID were requested by the parents or legal guardians. They also disclosed that informed consent was obtained in all cases from parents. All doctors refused to try less invasive options for this group of women.

### Biomedical aspects

All doctors (surgeons in particular) advocated that hysterectomy should be done for women with ID, where management of menstrual hygiene is a problem. To illustrate:

“*Hysterectomy should be performed only on those for whom menstrual hygiene is a problem or is anticipated to be a problem.”* (55).

“*Hysterectomy could be particularly relevant in low-income countries where poor socio-economic status might be an obstacle for long-term hormonal medication.”* (57).

Although all doctors (24, 54–57) perceived hysterectomy as a safe procedure from the point of view of no intra-and-post-operative morbidity, all these studies are not large enough nor long enough to examine rare or delayed operative consequences of hysterectomy. Of the five surgical studies, three omitted the histopathologic post-operative reports (24, 54, 56); and two mentioned that the histopathologic post-operative report was “uterus healthy” (55, 57). Furthermore, in none of the studies surgeons monitored the ovarian function after hysterectomy. Doctors (54–57) reported that parents/carers expressed satisfaction with the surgical results; while Piña-Perales (24) asserted that the quality of life patients' families improved. None of the authors, Kaunitz (55), Sheth (56), Piña-Perales (24), and Perrin (54) mentioned the research methodology that allowed them to draw these conclusions. In fact the participants' postoperative quality of life was not assessed by any of them. Chalermchockcharoenkit (57) used a Likert-type scale to assess satisfaction, 3 months after surgery, through telephone interviews. The carers expressed a high level of satisfaction with the outcome of the surgery (57).

### Overarching narrative synthesis of the entire dataset and development of a theoretical framework

The process began with privileging factors influencing the performance of hysterectomy for menstrual hygiene in women with ID as outlined in Figure 2; the factors were then used as the basis for developing a theoretical framework (Figure 3). The logic of the theoretical framework is described in the following paragraph:

Evidence suggests that relevant actors' reasons to request hysterectomy for menstrual hygiene constitute important ethical concerns in Public Health. In Figure 3 this notion is depicted in the upper left-hand square. These concerns influence the achievement of the fundamental objectives of Public Health: the creation and promotion of population health, as a broadly organized social response; the equal distribution of opportunities to be healthy (access to healthcare, education, information, material resources of various kinds); and the improving health-related autonomy. So, the upper right-hand square represents, theoretically, the realm of Public Health. The evidence also suggested which non-healthcare determinants of health would be important for the achievement of fundamental objectives of Public Health in this particular context. The link between the realms of non-healthcare determinants of health and healthcare system performance are core attributes of healthcare that increase the likelihood of achieving the desired outcomes: the need to restrict such surgical procedure, by analytically balancing benefits and burdens, in order not to reduce quality of life on the basis of arbitrary and partial judgments of personal preference or of social worth.

## Discussion

It appears that there is a worldwide move to harmonize the rights of women with ID with the Universal Declaration on Human Rights, by considering their capacity to make informed decisions or allowing parents and doctors arrive at what is the decision that is on the best interests of women with ID. However, to determine what constitutes respect for autonomy (capacity to make informed decisions) and the best interest course of action from various actor perspectives reveals the existing inequities.

For parents/carers, in general, menstrual hygiene problems were the main reason for requesting hysterectomy, particularly when the ID was severe. The request was related to parental distress, fears and anxieties, and the belief that personal hygiene training is unnecessary and laborious (13, 44–46, 48, 51, 52). Parental assumptions and concerns may gain support from Emerson's findings (10). He carried out a survey-based study, in the United Kingdom, with 9,481 children without ID and 245 children with ID, both groups aged 5–15 years. The information was collected by interviews with the primary carers and with some of the children. He found that families supporting children with ID are at increased risk of becoming socio-economically disadvantaged, and of becoming one-parent families, when compared with families supporting a child who did not have ID. Also, among mothers of children with ID, possible psychiatric morbidity was associated with the self-assessed psychological and social impact of the child's difficulties, and with unhealthy family functioning, and indicators of socioeconomic deprivation. Moreover, in low-and middle-income countries there is an absence of any active involvement of the state in health-related matters or financial support given to the families of women with ID (12, 13, 53, 59). In these countries (despite the potential legitimacy of the claims of parents/carers, and the idea that a woman's best interests will be promoted because she will no longer have menstrual bleeding) the decision-making process is not limited to only those factors that concern the direct interests of the women in question but, instead, incorporates the interests of others (parents/carers), those which arguably have a measurable impact on the well-being of the women with ID. On this matter, Beauchamp and Childress (60) state that sometimes values are incomparable or incommensurable and priority must be given to not directly harming one person, even though that will impose burdens on others.

For doctors who defend hysterectomy for menstrual hygiene, the respect for autonomy was understood as solely having to follow the parents/carers preferences (24, 54–57). They justified the surgical procedure by saying “*informed consent was obtained in all cases from parents/carers.”* In this regard, it is important recognise that there are facts that can distort parents/carers' judgment and limit the range of their autonomy; for instance, the great emotional distress along with the social and material burden of the care of a woman with ID (21). Consequently, to consider respect for autonomy as if it were only a right perpetuates the mistaken belief that respect for autonomy is a single state and something that can be achieved or conceded. To respect autonomy is basic to healthcare but it means to help women with ID and/or their parents/carers to exercise a more fully reasoned choice (60); otherwise it makes little sense to say that this is respect for autonomy.

In the cases of mild and moderate intellectual disability, some authors asserted that the consent or assent of the women in question is enough to perform hysterectomy (61). They fail to provide data that can illuminate how much women with ID understood the consent/assent process. Neither do they pay attention to the way they present the information to women. In this regard, Beauchamp and Childress (60) show that choices between risky alternatives can be heavily influenced by whether the same risk information is presented as providing a gain or an opportunity, or as constituting a loss or a reduction of opportunity. In addition, women with ID, even those ones with mild ID, could be immature or easily coerced. Therefore, it is naïve to assume that they could have exercised the right to self-determination.

The fact that the doctors perceived hysterectomy as a safe procedure from the point of view of the surgical technique and the peri and intra-operative morbidity was another important finding. The doctors reported that with hysterectomy they improved the “quality of life” of women with ID by eliminating menses permanently. Thereby, to doctors, particularly surgeons, the success of treatment was quantifiable, and the permanent elimination of menstrual bleeding and unwanted pregnancy were tangible benefits, with practically no intra-operative or post-operative morbidity. Within this approach to the decision, the medical/biological aspects underpin the doctors' choice. Satisfaction rates with hysterectomy among parents/carers were reported, by doctors, as very high (54–57). In fact, the studies included in this review reported strong parental approval for the hysterectomy outcomes. These findings provide several points that warrant discussion.

First, sound research in hysterectomized women aged 39 through 60 years (with ovarian conservation) show, especially in those aged 39 to 41 years, a significantly higher level of atypical climacteric complaints (tenseness, palpitations, irritability, etc.) together with a higher prevalence of the typical complains (flushes, sweating, and vaginal dryness) than in normal climacteric women of the same age (62). Observational studies report that hysterectomy (even with ovarian conservation) in premenopausal women is associated with significant loss of ovarian hormones, the ovarian function declination results in menopause several years earlier than average (63, 64). This is associated with a higher chance of developing coronary heart disease, neurological impairment, and increased risk of death from all causes (64, 65). Therefore, hormone replacement therapy becomes necessary (66); however, oestrogens will mitigate some but not all of these consequences (65). The long-term use of hormone replacement therapy has been linked to an increased risk for breast cancer, cardiovascular events, and stroke (67). All of these issues do not appear to have been monitored for women with intellectual disabilities.

Second, as for satisfaction before and after hysterectomy, it is impossible to establish exact figures for prevalence or incidence. In premenopausal women without ID, some observational studies show a very high rate of satisfaction 1 year after undergoing hysterectomy, as it relieves of abnormal bleeding and menstrual pain (68). Whereas, a randomized controlled trial that compared the effect of hysterectomy vs. expanded medical treatment on health-related quality of life, at the end of 2-year follow-up period found between-group differences that were not statistically significant in quality-of-life improvements (69). Extensive use of hysterectomy (in premenopausal women without ID) for benign gynaecological conditions has lead to discussions on the merits of this procedure.

And third, quality of life judgments require defensible criteria of benefits and burdens in order not to reduce quality of life to arbitrary judgements of personal preference and the patient's social worth (60). Our review shows that the studies reporting “quality of life improvements” (in women with ID that underwent hysterectomy) did not describe the research methodology used by the authors that support this assertion. Their quality of life criterion rests on the fact that the eliminating of menstrual bleeding and the non-morbidity of hysterectomy can decrease the burden of menstrual care to parents/carers. It is absolutely necessary that doctors not to confuse quality of life for the woman with ID with the satisfaction of carers or the value of that woman's life for others. As noted above, hysterectomy entails life-threatening conditions; hence, it is not proper to impute altruism or any other motive to that woman against her welfare.

The review of the findings indicated the intervention of some social factors in the hysterectomy request, in high income countries: life experiences and attitudes toward menstruation, and influence how the menstrual hygiene “problem” is managed (46, 47). Whereas in low-and middle-income countries, the intervening social factors were: socio-economic level (13), the availability of appropriate support (13, 24, 52, 53), the chance to have training and education about menstruation, and impact upon how menstruation is experienced by carers (13, 24, 53, 56). Many studies with women with ID moderate or severe have demonstrated that menstrual care skills can be taught to these women and that the skills were transferred from simulated to natural conditions. The skills learned persisted for 5 months (70), for 30 weeks (71), and for 18 months (72), in the natural environment. It is important to highlight that education is a strategy based on human rights and on widely accepted ethical principles against all forms of discrimination. It enables increasingly more women with ID to manage their own menstrual care independently. In a cross-sectional questionnaire survey with 452 carers of women with ID, in England, Rodgers (49) found that the level of independence of menstrual care was not associated with level of ID nor with age, but with the lack of educational opportunity and training appropriate to women' needs.

The practice of non- therapeutic hysterectomies in women with ID (even only in those with ID severe or profound) creates ethical concerns along a continuum; i.e., from considering the “quality of life” of the woman with ID to consider the value of this woman for society; from hysterectomy in post menarche women to hysterectomy in pre-menarche children (in some cases even without considering their level of ID); from performing the hysterectomy with the surgical techniques accepted for practice to the use of new and non-validated modalities of surgical techniques. Such is the case of Mexico, where there are no specific health policies and programs for people with ID. Although there exist 557 civil groups throughout the country that treat people with ID, 70% lack of financial resources, 47% lack healthcare professionals (12). On analysing the characteristics of those civil groups per socio-economical regions, it is shown that the worse figures for human and financial resources were gathered from the poorer regions of the country (12, 59). None of these regions have teaching programs for menstrual hygiene training. In these circumstances, it is not surprising that women with ID support interventions (hysterectomy) to suppress or eliminate menstruation. In the study by Marquez & Valdez (2018, Gaceta Medica de Mexico, in press); for example, hospitals belonging to the Social Security System are performing non- therapeutic hysterectomies in young women with ID, the majority of whom are moderately and severely/profoundly disabled. This surgical procedure is seen as morally acceptable by healthcare professionals; and therefore, it is proposed by them as a safe procedure for achieving menstrual hygiene. The paramount ethical issue here lies in the fact that the ethical quality of the actions depends, at least in part, upon the value of what they bring about.

## Limitations

Most of the empirical research about experiences and attitudes of the different actors (women with ID, parents/carers, doctors) toward non-therapeutic hysterectomy for menstrual hygiene is based primarily upon interviews with parents and carers; and to lesser extent, medical record reviews, and a case note review –this last one from clinical and legal notes. The majority of parents (*n* = 278/307, 91%) were natural mothers, and most of carers were female (*n* = 1455/1604, 91%). Only one study included the words of the women with ID. Five doctors reported quantitatively their personal series of cases of non-therapeutic hysterectomy in women with ID. Of the 17 studies, 11 were carried out in high income countries (US, UK, Australia; Canada) and with English speakers; and six in middle-income countries (India, Thailand, Taiwan and Mexico). Canada, Australia; US, and UK, have enacted legislation for sterilization of women with ID (21); hence, in all cases, a request for authorization perform a hysterectomy to cope with menstrual hygiene must be submitted to the Court. Nonetheless, this review showed that even in those developed countries, parental concerns remains a live issue, and they still request surgical control of menstruation.

The single study that did include the views and experiences of women with ID provides a snapshot of how they were positioned in the decision-making process. Furthermore, the fact that the voices heard are mainly those of parents/carers and doctors draws attention to the woman with ID as a voiceless participant in a medical and social reality in which her body becomes invisible to everyone else but her, rather than that instrument that makes her visible as the vulnerable human in need of protection that she is–even if the intention is to make a contribution to her individual good.

Although technically challenging and varying in quality all of the studies were considered to be of reasonable quality to include in the review. All, except four, were quantitative studies in design; and none of them reported sample size for quantitative studies or data saturation for qualitative studies thereby indicating the need for better designed studies in the future. Sounder empirical and long-term work is therefore needed to find out delayed operative consequences of hysterectomy; and to explore quality of life, after hysterectomy, in this particular group of women. Another important area in future research will be to increase involvement of the women with ID in the research.

This review provides evidence for room for further action in health policy initiatives to provide ethically sound healthcare to women with ID, in low-and middle-income countries. It also provides valuable evidence of the need for further educational programs to healthcare workers. The programs should enable these workers with the ability to work as a team with the women with ID and their families to make the rational and responsible choice that fits each woman. The programs should be case-based, and preferably the cases are those of real patients, families and providers. The findings reported have, therefore, important implications for policy and practice regarding menstruation and menstrual care for women with ID.

## Conclusions

The international ethical guidelines suggest that non-therapeutic hysterectomy in women with ID should not and ought not to be recommended as the routine and appropriate method to cope with menstrual hygiene even if it is technically safe. The results also showed that hysterectomy to cope with menstrual hygiene is still a live issue in high-, middle-, and low-income countries. In high income countries, non-therapeutic hysterectomy is performed with authorization from the Court, while in low-and middle-income countries there is not an active involvement of the State, or financial or training support for women with ID and their parents or their carers. Although, parents' informed consent was at the forefront of doctors' minds, before the hysterectomy performance, this was considered only as something necessary and achievable. In the end, the “problem” with menstrual hygiene in women with ID, in LMIC, cannot be solved by a selective choice of certain values over others, but, rather, by investigating the options available, and incorporating a range of alternatives, supports and resources along with the education and training for the women concerned, their parents and their caregivers, followed by further training of all professionals involved. This will provide more justice for the women and this means better and fairer choices for all involved, including that of the community in general. Hence, in low-and middle-income countries there is an urgent need to develop and enact policies and statutes in this area of public health and clinical practice.

## Author contributions

EV-M and HM-G were responsible for review questions and design, conducted hand search of the literature and supervised the electronic search, conducted the quality appraisal, and undertook the analysis. EV-M developed the theoretical framework. EV, HM-G, and MB made critical revisions to the paper and refined the ethical ideas that support the conclusions proposed. EV-M coordinated all process of data collection and supervised the study. All authors read and approved the final paper.

### Conflict of interest statement

The authors declare that the research was conducted in the absence of any commercial or financial relationships that could be construed as a potential conflict of interest.
